# Macrophages Are Key Regulators of Stem Cells during Skeletal Muscle Regeneration and Diseases

**DOI:** 10.1155/2019/4761427

**Published:** 2019-07-14

**Authors:** Junio Dort, Paul Fabre, Thomas Molina, Nicolas A. Dumont

**Affiliations:** ^1^CHU Sainte-Justine Research Center, Montreal, QC, Canada; ^2^School of Rehabilitation, Faculty of Medicine, Université de Montréal, Montreal, QC, Canada; ^3^Department of Pharmacology and Physiology, Faculty of Medicine, Université de Montréal, Montreal, QC, Canada

## Abstract

Muscle regeneration is a closely regulated process that involves a variety of cell types such as satellite cells, myofibers, fibroadipogenic progenitors, endothelial cells, and inflammatory cells. Among these different cell types, macrophages emerged as a central actor coordinating the different cellular interactions and biological processes. Particularly, the transition of macrophages from their proinflammatory to their anti-inflammatory phenotype was shown to regulate inflammation, myogenesis, fibrosis, vascularization, and return to homeostasis. On the other hand, deregulation of macrophage accumulation or polarization in chronic degenerative muscle disorders was shown to impair muscle regeneration. Considering the key roles of macrophages in skeletal muscle, they represent an attractive target for new therapeutic approaches aiming at mitigating various muscle disorders. This review aims at summarizing the novel insights into macrophage heterogeneity, plasticity, and functions in skeletal muscle homeostasis, regeneration, and disease.

## 1. Introduction

Skeletal muscle injury can be caused by a variety of conditions such as direct trauma, disuse, ischemia, exercise, toxins, and genetic diseases. To face these challenges, skeletal muscle has developed a remarkable regenerative capacity, which relies on muscle stem cells, named satellite cells. Skeletal muscle regeneration is a tightly regulated process during which quiescent satellite cells are activated and become proliferating myoblasts, which will differentiate and fuse to form multinucleated myotubes (newly formed muscle fiber) [1]. The coordination of the myogenesis process (formation of new muscle tissue) involves the cooperation of numerous other cellular and molecular components [2]. Particularly, the onset, development, and the resolution of the inflammatory response play an instrumental role in the regulation of myogenesis.

Monocytes and macrophages are predominant myeloid cells that chronologically accumulate in skeletal muscle at the onset of injury-induced inflammation [3]. There are numerous evidences indicating that macrophages are key regulators of different biological processes involved during skeletal muscle regeneration, such as myogenesis, fibrosis, inflammation, and revascularization [3–9]. On the other hand, in chronic degenerative conditions, the excessive and disorganized influx of macrophages stimulates muscle necrosis, fibrosis, and defective muscle repair. Therefore, the spatiotemporal regulation of inflammation is vital for an effective regeneration of skeletal muscle.

In recent years, novel discoveries revealed that the plasticity, heterogeneity, and the roles played by macrophages in skeletal muscles are much more complex than anticipated. This review will discuss these novel insights into the role of macrophages in muscle homeostasis, regeneration, and diseases with a particular focus on Duchenne muscular dystrophy (DMD). Promising strategies targeting macrophage polarization in physiopathological conditions will also be discussed.

## 2. Origin and Recruitment of Monocyte and Macrophages

Numerous tissues contain long-lived resident macrophages that originate from the yolk sac during development [10]. In steady state, these tissue-resident macrophages self-renew through in situ proliferation or are replenished by blood monocytes [11–13]. Resident macrophages are observed in healthy skeletal muscles where they regulate tissue homeostasis. In rats, resident macrophages are identified by the marker ED2, while infiltrating monocytes/macrophages are defined by the expression of the marker ED1. In humans, resident macrophages were shown to largely coexpress CD11b and CD206 [14]. Contrary to infiltrating macrophages, ED2^+^ resident macrophages do not contribute to phagocytosis [15]; instead, it is suggested that they act as sentinels that are readily activated by damage-associated molecular patterns (DAMPs) secreted during muscle injury to facilitate the invasion of circulating leukocytes. However, the literature on these resident cells is limited, and further research is needed to clearly comprehend their roles in healthy and regenerating skeletal muscle.

After an injury, activated monocytes originating from the bone marrow adhere to the blood vessels, roll, and migrate to damaged sites, where they start differentiating into macrophages. In mice, two main monocyte subsets have been described according to their mechanism of extravasation and their level of expression of the protein Ly6C [16, 17]. The proinflammatory Ly6C^hi^ population recruited via the C-C motif chemokine receptor 2 axis (CCR2/CCL2) preferentially accumulates during the acute phase of inflammation, while the CX3C chemokine receptor-1- (CX3CR1-) dependent Ly6C^lo^ subset appears later and exhibits anti-inflammatory properties. Similar monocyte subsets have also been identified in humans using the markers CD14 and CD16. Monocytes CD14^hi^CD16^lo^ correspond to the Ly6C^hi^ monocytes in mice, while CD14^lo^CD16^hi^ relate to the Ly6C^lo^ monocyte profile [16].

The mechanism of monocyte recruitment appears to be specific to the tissue and the nature of the insult. For instance, both Ly6C^hi^ and Ly6C^lo^ were shown to sequentially invade the injured tissue after myocardial infarction using their CCR2 or CX3CR1 receptor, respectively [18]. On the other hand, it has been shown that only the Ly6C^hi^ subtype is recruited during sterile skeletal muscle injury, which thereafter switch to the Ly6C^lo^ phenotype [17]. The phagocytosis of apoptotic neutrophils by macrophages was shown to partially contribute to this switch [17]; however, it is likely that many other cellular and chemical interactions present in the dynamic regenerative microenvironment also contribute to this process. In addition to their transition from Ly6C^hi^ monocytes/macrophages, the Ly6C^lo^ cells also accumulate from local proliferation [19]. This finding was also observed in rats where the accumulation of ED1^+^ and ED2^+^ macrophages was shown to be partially mediated by local proliferation, especially when invasion of circulating monocytes is reduced by injection of liposome-encapsulated clodronate [20]. Notably, while the different subsets of macrophages were suggested to accumulate sequentially in the injured tissue, it is important to notice that both subsets of macrophages could be simultaneously present in acute regenerating muscles [21], a phenomenon which is exacerbated in chronic degenerative muscle diseases such as DMD [22].

## 3. Macrophage Subsets and Polarization

A general classification suggests that macrophages can be immunologically classified into two main subsets according to their specific functions: the “classically activated” M1 macrophages, which are present in the inflammatory period and associated with phagocytosis, and the “alternatively activated” M2 macrophages, accumulating at the site of injury once necrotic tissue has been removed and participating in the regeneration and remodelling process. *In vitro*, the M2 phenotype has been further classified into three main subsets—M2a, M2b, and M2c—each of which requires specific polarization cues [23, 24]. The alternatively activated M2a macrophages arise from exposure to interleukin-4 (IL-4) and IL-13, the M2b subtype is polarized by IL-1 receptor ligands, and the M2c phenotype is promoted by IL-10 and glucocorticoids [25, 26]. In mice, the M2 macrophages are identified by the expression of the pan-macrophage marker F4/80 and the alternative activation markers such as Fizz-1 and Ym1 [27]. Of note, Arginase-1 was considered as a specific marker for M2 macrophages; however, it is also expressed in the spectrum of M1 macrophage polarization [28]. In humans, M2 macrophages express the pan-macrophage marker CD68 and alternative activation markers such as CD163 and/or CD206 [27].

Recent insights suggest that this classification based on specific activating factors *in vitro* is an important underestimation of the different macrophage subsets. Accordingly, a study investigating the transcriptional program of macrophages showed that there is a wide spectrum of macrophage activation states [29]. The authors showed that while the bipolar activation state is maintained when the macrophages are stimulated with factors classically associated with M1 or M2 polarization (e.g., TNF-*α* vs. IL-4), it becomes much more complex when other factors such as fatty acids or a combination of molecules associated with chronic inflammation are used. From the 29 different conditions tested, the authors identified 10 major clusters of activation [29]. These different conditions *in vitro* only give a glimpse of the complexity of the microenvironment of macrophages during muscle regeneration *in vivo*. Indeed, macrophages are interacting with a fluctuating network of hundreds of different molecular, physical, and cellular components that affect their phenotype. For instance, after their extravasation into the injured tissue, monocytes will attach to the extracellular matrix (ECM), which continuously evolves during muscle regeneration. Notably, components of the ECM, such as collagen and fibrinogen, were shown to stimulate macrophage phagocytosis and expression of proinflammatory factors, respectively [30]. Alternatively, attachment of macrophages through their *α*4*β*1 integrin receptor to ECM matrix components (fibronectin and vascular cell adhesion molecule-1 (VCAM-1)) stimulates their transition toward the anti-inflammatory phenotype by activating Rac2 signalling [31]. Moreover, macrophage polarization is also sensitive to mechanical stress. Low-frequency mechanical stretch pushes macrophages toward the anti-inflammatory phenotype, while high-frequency strains maintain macrophages in their proinflammatory state [32]. These results suggest that macrophage activation and polarization *in vivo* are processes that are much more complex than what has been described so far. Accordingly, recent analysis of macrophage transcriptional signature after an *in vivo* muscle injury induced by cardiotoxin showed that Ly6C^hi^ and Ly6C^lo^ macrophages only partially overlap with M1 and M2 gene expression patterns, respectively [19]. Instead, the authors showed that the time lapse was the prevalent driving force regulating macrophage gene expression profile, suggesting that a global and coordinated change in the microenvironment components is required to regulate macrophage polarization. The authors identified four key features that account for the changes in gene expression in macrophages during the course of muscle regeneration: firstly, an early expression of genes involved in acute inflammation (e.g., S100A8/9, lipocalin-2, haptoglobin, formyl peptide receptor-1, and leukotriene B4 receptor-1); secondly, a metabolic shift from glycolysis to glutamine and oxidative metabolism-associated genes (e.g., glutamate synthase-1, glycerol-3-phosphate dehydrogenase 2, and superoxide dismutase 2); thirdly, a transient increase in genes associated with cell proliferation (e.g., cyclin-D1 and -A2, many members of the minichromosome maintenance (mcm-2, -3, -4, -5, -6, and -7), DNA ligase-1, replication factor C subunit-1, and ribonucleotide reductase catalytic subunit-M1 and -M2); and fourthly, an increase in the expression of ECM genes (e.g., fibrillin-1, decorin, periostin, lumican, osteonectin, and biglycan). These different clusters of genes could be used to identify new markers to characterize macrophage heterogeneity in skeletal muscle regeneration.

Overall, the M1 and M2 macrophage nomenclature is oversimplistic to characterize macrophage polarization *in vivo*, which should rather be considered as a continuum of activation. Recent effort has been made to propose a common framework for the macrophage-activation nomenclature [28]. Here, for the sake of clarity, we propose to use a bipolar nomenclature (proinflammatory vs. anti-inflammatory) to describe the two opposite sides of the spectrum of macrophage activation; however, one should keep in mind that the actual activation state of macrophages is much more plastic, heterogeneous, and complex.

## 4. Macrophages Regulate the Different Biological Processes Implicated in Acute Skeletal Muscle Healing

### 4.1. Macrophages Interact with Other Leukocytes to Regulate Inflammation

**Figure 1:**
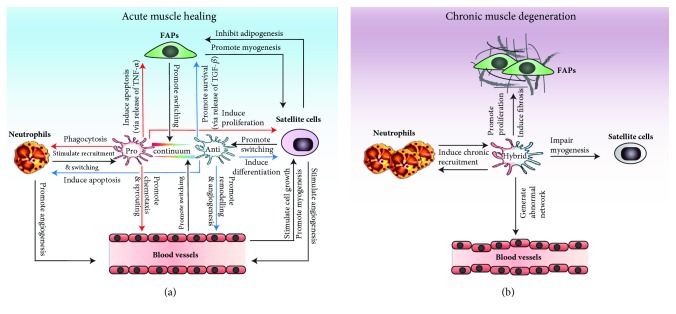
Macrophages are central regulators in skeletal muscle regeneration and diseases. In acute muscle injury (a), the inflammatory process is characterized by early accumulation of proinflammatory macrophages, which play a key role in various biological processes involved in muscle regeneration, by regulating fibrosis (FAP apoptosis), myogenesis (satellite cell proliferation), angiogenesis (sprouting), and inflammation (phagocytosis). Thereafter, macrophages switch toward the anti-inflammatory phenotype, which dampens inflammation, stimulates satellite cell/myoblast differentiation, and promotes tissue remodelling. This temporal and coordinated process is essential for optimal muscle healing. In a chronic degenerative muscle (b), the concurrent pro- and anti-inflammatory signals lead to the adoption of an abnormal hybrid phenotype by macrophages, which promote chronic inflammatory cell infiltration, excessive fibrosis, impaired myogenesis, and disorganized blood vessel network.

The inflammatory process is constituted of different types of leukocytes such as mast cells, neutrophils, eosinophils, monocytes/macrophages, and lymphocytes, which have all been shown to act on skeletal muscle regeneration [6]. Particularly, monocytes/macrophages emerged as the key cellular component orchestrating leukocyte accumulation and function during the different phases of the inflammatory process, i.e., the onset, development, and resolution stages (Figure 1).

#### 4.1.1. Onset

As described previously, resident macrophages are important to sense damage to the tissue and initiate the recruitment of circulating leukocytes. Once activated, resident macrophages secrete chemokines such as cytokine-induced neutrophil chemoattractant 1 (CINC-1) and monocyte chemoattractant protein-1 (MCP-1) that promote the recruitment of neutrophils and monocytes [6]. Moreover, it was also observed that a subset of Ly6C^lo^ circulating monocytes was “crawling” inside the blood vessels independently of the blood flow, with the help of their receptors CX3CR1 and lymphocyte function-associated antigen-1 (LFA-1) [33]. These patrolling monocytes sense tissue damage or infection and transiently invade the tissue as soon as 1 h after an insult (much faster than other circulating leukocytes). At this timepoint, patrolling monocytes are the principal source of TNF-*α*, which promotes the recruitment of other inflammatory cells. Moreover, patrolling monocytes were also shown to promote the recruitment of neutrophils through prolonged cell-cell contact in the microvasculature [34]. This direct physical interaction stimulates neutrophil retention and production of reactive oxygen species (ROS) at the site of the injury.

#### 4.1.2. Development

Starting a few hours after the injury, the accumulation of neutrophils in the injured muscle remains elevated for a few days. Neutrophils stimulate host defense and the clearance of cell debris by phagocytosis and by the release of ROS and proteases [3]. Accordingly, depletion of neutrophils during acute muscle regeneration leads to persistence of necrotic tissue and delayed regeneration [35]. Moreover, a subset of neutrophils was also shown to promote angiogenesis [36]. Neutrophils also stimulate the development of the inflammatory process by expressing cytokines such as macrophage inflammatory protein 1 (MIP-1*α*) and MCP-1 that attract circulating monocytes at the damaged sites [37]. Ly6C^hi^ monocytes massively infiltrate in the injured muscle, where they play a key role in the development of inflammation by secreting proinflammatory cytokines such as TNF-*α* that further promote the recruitment of neutrophils and monocytes [17, 38]. This proinflammatory environment peaks around 48 h after the injury. Thereafter, Ly6C^lo^ monocytes become the predominant subsets in the regenerating muscle, in which they play a key role to dampen inflammation.

#### 4.1.3. Resolution

The phase of resolution of inflammation is not a passive process caused by the decrease in proinflammatory signals; it is an active process that involves a variety of cell types and mediators [39]. Ly6C^lo^ antimacrophages are actively promoting the resolution of inflammation by expressing a wide array of anti-inflammatory cytokines (e.g., IL-4 and IL-13) and by switching their expression of proinflammatory lipids (e.g., prostaglandin-E_2_ (PGE_2_)) to proresolving lipids (e.g., 15*Δ*-PGJ2) [40]. These mediators do not only reduce proinflammatory signals and ROS production, but they also actively stop the recruitment of neutrophils and promote their apoptosis and their nonphlogistic phagocytosis by macrophages [39]. Accordingly, the depletion of macrophages during muscle regeneration prolonged the presence of neutrophils in the injured muscle [41]. The importance of macrophages in the resolution of inflammation is crucial considering that the chronic presence of inflammatory cells has been associated with impaired tissue regeneration. At the late stages of muscle regeneration, macrophages ceased the expression of both pro- and anti-inflammatory cytokines and turned to a silenced mode, which precede the return to homeostasis [42]. Overall, monocytes/macrophages play a central role in the regulation of inflammation from the beginning to the end.

### 4.2. Macrophages Interact with Satellite Cells to Regulate Myogenesis

Proinflammatory macrophages are key regulators of the host defense, and they are typically associated with clearance of cell debris during skeletal muscle repair [3, 8]. Necrotic fibers may act either as atrophic factors to repress myoblast growth or as physical barriers to prevent myoblast contact, indicating that sufficient infiltration of macrophages might be required for proper regeneration. For instance, using a mouse model deficient in CCR2, which is essential for Ly6C^hi^ monocyte extravasation, it was shown that the drastic reduction of infiltrating monocytes following muscle injury induced by ischemia [43], notexin or cardiotoxin [17, 44], and barium chloride [45] is accompanied by altered muscle regeneration. This impaired regeneration was partially mediated by insufficient phagocytosis of necrotic fibers [45]. However, even after adequate phagocytosis, myofibers failed to efficiently recover when intramuscular macrophages are depleted in a model of notexin-induced injury in mice [17].

Macrophages have multiple beneficial roles during muscle regeneration in addition to their participation in the clearance of cell debris. Particularly, the importance of macrophages in the regulation satellite cells and myoblasts during the myogenesis process is now well defined (Figure 1). The hypothesis that macrophages promote myogenesis was first supported by experiments showing that macrophage-conditioned medium triggers myoblast proliferation *in vitro* and improves muscle regeneration *in vivo* [46, 47]. The crucial role of macrophages to stimulate myogenesis is further illustrated in a model of 3D muscle construct *in vitro*, in which the addition of macrophages is necessary to allow the tissue to self-repair after an injury [48]. Pioneer work from Chazaud's lab has shown that the release of proinflammatory cytokines by Ly6C^hi^ macrophages promotes myoblast proliferation and inhibits differentiation, while the release of anti-inflammatory cytokines by Ly6C^lo^ macrophages inhibits myoblast proliferation and stimulates their differentiation and fusion [17]. The exact cocktail of paracrine factors regulating satellite cell function has not been precisely characterized; however, many molecules secreted by macrophages have been shown to partially mediate these effects. For instance, cytokines highly expressed by proinflammatory macrophages such as interleukin-6 (IL-6) [49], TNF-*α*, and PGE_2_ [50] were shown to stimulate satellite cell proliferation. Moreover, Ly6C^hi^ macrophages secrete the enzyme ADAMTS1 (A Disintegrin-Like And Metalloproteinase With Thrombospondin Type 1 Motif) that reduces the Notch signalling pathway, leading to increased satellite cell activation and muscle regeneration [51]. On the other hand, cytokines and growth factors highly expressed by anti-inflammatory macrophages such as interleukin-4 (IL-4) [52] and insulin-like growth factor-1 (IGF-1) [41] were shown to stimulate myoblast differentiation/fusion and myofiber growth. In addition to paracrine factors, the direct physical contact of myogenic cells with macrophages is important to regulate their cell function and fate decision. Accordingly, *in vitro* coculture of macrophages and myogenic cells showed that macrophages have a proproliferative effect through the release of paracrine factors and an antiapoptotic effect by direct physical contact through a set of different adhesion molecules (VCAM1, intercellular adhesion molecule-1 (ICAM-1), platelet endothelial cell adhesion molecule-1 (PECAM-1), and CX3CR1) [53, 54].

The critical role of the different subsets of macrophages was also confirmed *in vivo*. It was first observed that in regenerating muscle, proinflammatory macrophages are in close proximity to proliferating satellite cells, while anti-inflammatory macrophages are near to the regenerating area containing differentiated myoblasts [21]. Depletion experiments were used to further characterize the role of the different subsets of macrophages *in vivo*. For instance, the depletion of infiltrating Ly6C^hi^ monocytes using genetic models or pharmacological compounds, prolonged the presence of necrotic cells, promoted the accumulation of muscle fat and fibrosis, and impaired the overall muscle regeneration [17, 41, 55, 56]. On the other hand, the suppression of the ability of macrophages to switch to their anti-inflammatory phenotype, induced by loss-of-function mutations in AMP-activated protein kinase-1 (AMPK*α*1) [57], IGF-1 [58], CCAAT/enhancer binding protein-*β* (CEBPB) [59], or peroxisome proliferator-activated receptor-*γ* (PPAR-*γ*) [60], was shown to reduce muscle fiber growth, without affecting the removal of necrotic tissue. In turn, models of satellite cell deletion also showed to have delayed macrophage transition to their anti-inflammatory phenotype, suggesting that there is a regulatory feedback by which myogenic cells contribute to the phenotypic switch of macrophages [61]. Altogether, these *in vitro* and *in vivo* experiments demonstrate that the different subsets of macrophages have complementary roles in the regulation of satellite cell/myoblast function, myogenesis progression, and optimal muscle regeneration. Furthermore, these findings also suggest that the temporal and spatial recruitment of macrophages is crucial to regulate the progression of satellite cells through the myogenesis process. Therefore, disorganized macrophage accumulation could send aberrant signals to satellite cells and impair their myogenesis capacity, which will be further discussed later in this manuscript.

### 4.3. Macrophages Interact with FAPs to Regulate Muscle Fibrosis

Another stem cell type, the fibroadipogenic progenitors (FAPs), plays a crucial role in skeletal muscle regeneration. These tissue-resident stem cells can differentiate into fibroblasts or adipocytes. In acute skeletal muscle injury, FAPs support satellite cell activation and differentiation and, retroactively, satellite cells inhibit FAP differentiation into adipocytes [62–64]. However, in chronic muscle disorders, FAPs can turn into direct contributors of ectopic fat deposition and formation of fibrotic scars that fail to support satellite cell activity [65]. Therefore, FAP activity and accumulation need to be closely regulated. It was shown that FAPs quickly and massively accumulate in the early phase of acute muscle injury, while their number quickly decreases after a few days [5]. Interestingly, this decrease in FAP accumulation correlates with the peak of macrophage accumulation [2]. It was demonstrated that the infiltration of proinflammatory macrophages is essential to control the accumulation of FAPs, via their secretion of TNF-*α* that directly stimulates FAP apoptosis [5]. Nitric oxide is another factor abundantly produced by proinflammatory macrophages that was shown to inhibit FAP differentiation toward adipocytes *in vitro* and to reduce the deposition of intramuscular fat and connective tissue *in vivo* [66]. The absence of monocyte recruitment to the site of injury in CCR2^−/−^ mice or following diphtheria toxin injection to ITGAM-DTR mice impairs FAP clearance and prolongs their presence in the injured muscle leading to abnormal collagen deposition [5, 67]. On the other hand, anti-inflammatory macrophages release transforming growth factor-*β* (TGF-*β*) that promotes FAP survival, which could be important for tissue remodelling during late muscle healing phases. Coculture of fibroblasts with the different subsets of macrophages confirmed that anti-inflammatory macrophages promote fibroblast proliferation and collagen synthesis, while proinflammatory macrophages reduce collagen synthesis and secrete enzymes such as MMP-1 and MMP-3 that degrade ECM [68, 69]. In turn, evidence suggests that a subset of FAPs could also contribute to the phenotypic switch of macrophages [67].

Overall, macrophages play a crucial role to control fibrogenic cell accumulation and activity and to regulate muscle fibrosis. Particularly, the sequential accumulation of the different macrophage subsets is decisive in this process to find the delicate balance that not only limits the excessive activity of fibrotic cells and fibrosis deposition but also allows tissue remodelling needed for the return to homeostasis (Figure 1).

### 4.4. Macrophages Interact with Endothelial Cells to Regulate Neovascularization

In steady state, satellite cells reside in close proximity to the blood vessels [70]. There is a regulatory cross talk by which satellite cells secrete VEGFA to recruit endothelial cells, which in turn maintain satellite cell quiescence through the notch ligand Dll4 (Delta-like 4) [70]. Similarly, the interaction between angiopoietin-1 secreted by the smooth muscle cells and the Tie-2 receptor of the satellite cells was also demonstrated to promote quiescence [71]. Following an injury, cells from the blood vessels interact with satellite cells to promote revascularization, which is critical for muscle recovery. Particularly, endothelial cells directly regulate satellite cell growth by secreting various growth factors (IGF-1, hepatocyte growth factor (HGF), basic fibroblast growth factor (bFGF), platelet-derived growth factor (PDGF), and vascular endothelial growth factor (VEGF)), and through a retroactive loop, differentiated myoblasts stimulate angiogenesis [72]. Similarly, pericytes, which are juxtaposed to capillary endothelial cells, were shown to have myogenic capacities *in vitro* and to stimulate myoblast function and muscle regeneration *in vivo* [73].

Macrophages play a central role during muscle regeneration to regulate the function of endothelial cells, which in turn promote the polarization of macrophages to their anti-inflammatory phenotype (Figure 1) [74]. For instance, the depletion of infiltrating monocytes in CCR2^−/−^ mice impairs collateral arteriogenesis after ischemic hindlimb occlusion [75]. However, the role of the different subsets of macrophages on vascularization is still debated. The proangiogenic role of tumour-associated macrophages, a distinct subset of anti-inflammatory macrophages [76], is well defined; however, the role of the different macrophage subsets in a nontumourigenic environment is variable and dependent on various factors. An *in vitro* study indicated that anti-inflammatory macrophages promote the formation of new blood vessels to a higher level than proinflammatory macrophages [77]. Another *in vitro* model showed that proinflammatory macrophages (stimulated with lipopolysaccharide (LPS) + interferon-*γ* (IFN-*γ*)) increase the length and number of blood vessel sprouts to a higher level than anti-inflammatory M2a macrophages (induced by IL-4 + IL-13), but to a lower level than anti-inflammatory M2c macrophages (induced by IL-10) [78]. Notably, the anti-inflammatory M2a macrophage subset in these experiments produced higher levels of paracrine factors recruiting pericytes [78]. A model of *in vitro* coculture between endothelial cells, myogenic progenitor cells, and macrophages stimulated with IL-4 or IL-10 showed that anti-inflammatory macrophages coordinate angiogenesis and myogenesis in part by the secretion of oncostatin M [79]. Overall, the subsets of macrophages play several roles that contribute to the different phases of angiogenesis. Accordingly, it was shown that the subsequent incubation of endothelial cells with proinflammatory followed by anti-inflammatory M2a macrophages *in vitro* (to mimic the macrophage phenotype switch observed *in vivo*) enhances the blood vessel network formation [78].

Neovascularization was studied *in vivo* with different models of biomaterial implementation. Similar to *in vitro* experiments, the conclusion regarding the roles of the different subsets of macrophages on angiogenesis is variable depending on the experimental design and the outcomes measured. While some studies indicated that anti-inflammatory macrophages are primarily responsible for microvascular network growth and remodelling [80], others showed that the vascularization is related to a higher ratio of proinflammatory : anti-inflammatory macrophages [81]. This discrepancy might be related to the diversity of macrophage phenotypes *in vivo* and to the complex regulatory network between these subsets of macrophages and the numerous cell types involved in angiogenesis. Overall, macrophages play a crucial role in the regulation of muscle revascularization after an injury; however, further studies are needed to delineate the specific impacts of the different subsets of macrophages.

## 5. Macrophages in Chronic Muscle Disorders

To mediate their beneficial effects on the different cellular processes involved in skeletal muscle regeneration, the accumulation of the different subsets of macrophages needs to be controlled, transient, and sequential. Disorganization or excessive macrophage activity is a common feature of many chronic conditions, which contributes to tissue degeneration. For instance, iron overloading caused by the excessive engulfment of erythrocytes by anti-inflammatory macrophages induces their switch to an unrestrained proinflammatory phenotype, which stimulates chronic inflammation and impairs wound healing [82]. Asynchronous muscle injuries (induced by two consecutive traumatic injuries separated by a few days) also perturb the proper course of inflammation leading to the concurrent (nonsequential) accumulation of proinflammatory and anti-inflammatory macrophages in the injured area that increases muscle fibrosis [83].

Many muscular diseases are associated with chronic inflammation and impaired muscle regeneration. For instance, skeletal muscles from patients with Pompe disease, which is caused by acid-alpha glucosidase deficiency resulting in lysosomal glycogen accumulation, are subjected to an excessive invasion of proinflammatory macrophages that is correlated with impaired satellite cell differentiation [84]. Similarly, dysferlinopathy, another type of progressive myopathy caused by a mutation in the dysferlin gene, is associated with chronic accumulation of macrophages. These macrophages are maintained in a cytodestructive proinflammatory state that promotes myogenic cell apoptosis/necrosis [85].

The most studied form of muscular disorders is DMD, a frequent and severe debilitating disease characterized by progressive muscle weakness resulting in loss of ambulation, respiratory dysfunctions, and premature death. DMD is caused by a mutation in the gene that encodes for dystrophin, a protein important for muscle fiber stability and for satellite cell function [86]. Therefore, in the absence of dystrophin the muscles are subjected to repetitive and overlapping cycles of degeneration and regeneration. The microenvironment in these dystrophic muscles is characterized by the overactivation of inflammatory pathways such as NF-*κ*B [87], increased cell membrane permeability, and abnormal intracellular calcium influx, as well as a deregulated nitric oxide signalling [88]. These abnormalities provoke changes in gene expression toward a chronic inflammatory molecular signature characterized by the high expression of molecules associated with cytokine and chemokine signalling, vascular adhesion and permeability, and lymphoid and myeloid markers [89]. Particularly, osteopontin is one of the most highly upregulated genes in muscles from mdx mice (mouse model of DMD) and in DMD patients [89, 90]. Osteopontin is an immunomodulator protein involved in immune cell migration and survival, and its ablation in dystrophic mdx mice was shown to promote the transition of proinflammatory macrophages toward their anti-inflammatory phenotype leading to reduced fibrosis and improved muscle function [91]. The chronic inflammatory environment in dystrophic muscles promotes the long-lasting recruitment of neutrophils and monocytes/macrophages, which instead of contributing to tissue clearance through phagocytosis of cell debris, rather stimulate muscle cell lysis [92] through their high levels of expression of cytotoxic molecules such as ROS. Accordingly, the depletion of neutrophils [93] or monocytes [92] reduces the number of necrotic fibers in mdx mice. Interestingly, the ablation of CCR2 in mdx mice not only reduces the number of infiltrating macrophages but also restores the macrophage polarization balance by skewing macrophages to their anti-inflammatory phenotype, which decreases muscle histopathology and increases muscle force [94]. This beneficial effect was not sustained at long term, potentially due to the local proliferation of resident macrophages that compensate for the lack of infiltrating monocytes [94, 95].

In contrast to the self-limited inflammation following acute sterile muscle injury, the conflicting signals sent simultaneously by degenerative and regenerative environments in chronic or excessive muscle injuries impair macrophage polarization. For instance, following a massive injury induced by muscle laceration, macrophages adopt an intermediary phenotype, which was associated with impaired muscle regeneration and persistent collagen deposition [96]. Interestingly, in this model, the exogenous transplantation of proinflammatory macrophages in the injured muscle reestablished the polarization state, which resulted in decreased fibrosis and improved muscle healing. Likewise, macrophages expressing high levels of both the proinflammatory macrophage marker iNOS (inducible nitric oxide synthase) and the anti-inflammatory macrophage marker CD206 have been observed in mdx mice [94]. In dystrophic muscles, hybrid macrophages expressing high levels of both proinflammatory cytokines (TNF-*α*) and anti-inflammatory cytokines (TGF-*β*) showed their inability to reduce the accumulation of FAPs in the injured muscle [5]. Similarly, it was shown that the binding of macrophages to excessive fibrinogen deposition in dystrophic muscle stimulates the production of the proinflammatory cytokine IL-1*β* together with TGF-*β* [97]. These hybrid macrophages, particularly Ly6C^hi^ macrophages expressing high levels of LTBP4 (latent TGF-*β* binding protein), promote the overexpression of the ECM component by FAPs and fibroblasts, leading to aberrant muscle fibrosis [98]. Interestingly, therapeutic strategies promoting the switch of macrophages toward the proinflammatory or anti-inflammatory phenotype were demonstrated to reduce fibrosis in dystrophic mice. For instance, blocking TGF-*β*-induced p38 kinase activation with the tyrosine kinase inhibitor Nilotinib restores the ability of proinflammatory macrophages to induce FAP apoptosis and promote the resolution of fibrosis in mdx mice [5]. On the other hand, skewing macrophages toward their anti-inflammatory phenotype by AMPK activation blocks their production of latent-TGF-b1 and reduces fibrosis deposition [98]. Overall, the chronic and deregulated macrophage accumulation and polarization observed in dystrophic muscles perturb the inflammatory process, enhance myofiber degeneration, impair myogenesis, and stimulate fibrosis deposition, which contribute to accelerate the progression of the disease (Figure 1).

## 6. Macrophages in Muscle Aging

Aging is associated with progressive degeneration that affects multiple tissues, including skeletal muscles. Progressive loss of muscle mass of approximately 1% to 2% per year is observed beyond the age of 50 [99]. In some conditions, aging is also associated with sarcopenia, a phenomenon characterized by progressive and generalized loss of muscle mass and force/function leading to physical disability, poor quality of life, and death. Genome-wide transcription analysis revealed that the expression of inflammatory- and immunology-related genes is particularly affected in skeletal muscle during aging [100]. Evidence suggests that altered macrophages during aging impair satellite cell function and muscle regeneration. An *in vitro* model showed that conditioned medium collected from old bone marrow-derived macrophages (BMDM) decreased the number of Ki67^+^ myoblasts compared to conditioned medium generated from young BMDM, suggesting a reduction in the ability of macrophages to secrete proproliferative factors during aging [101]. In resting muscles of aged mice, an increase in M2a macrophages (CD68^+^CD163^+^) has been observed, which correlates with an increase in skeletal muscle fibrosis [102]. Furthermore, the transplantation of bone marrow cells isolated from young mice into aged mice prevented the increase of M2a macrophages and the accumulation of connective tissues in these muscles. In humans, one study comparing young (21-33 years) to elderly subjects (70-81 years) showed that total macrophage density (CD68^+^) is not different between the two groups, but that the gene expression of CD206 is higher in the elderly group, suggesting an increase in the proportion of anti-inflammatory macrophages in aging human skeletal muscle, similar to what has been observed in mice [103]. However, another study showed that in elderly subjects (average 71.4 years), there is a decrease in the number of both proinflammatory macrophages (CD11b^+^ cells) and anti-inflammatory macrophages (CD163^+^ cells) when compared to young individuals (average 31.9 years) [104]. Notably, both subpopulations of macrophages increase following acute resistance exercise in young adults but not in the elderly, indicating an impaired ability of aged muscle to develop a coordinated inflammatory response. Moreover, another study investigating the effect of aging on skeletal muscle macrophages in different conditions (healthy, bed rest, and rehabilitation exercise) showed that elderly individuals (average 66 years old) have less proinflammatory macrophages (CD11b^+^CD68^+^) and a similar number of anti-inflammatory macrophages (CD68^+^CD163^+^) than young individuals (average 23 years old) in each condition [105]. These studies indicate that the effect of aging on skeletal muscle macrophages in humans is variable depending on the marker used, the population examined, and the condition studied. Overall, we can conclude that the function of macrophages in skeletal muscle homeostasis and regeneration seems to be perturbed during aging; however, further high-quality research is needed to better define these dysfunctions and comprehend the physiopathological mechanisms.

## 7. Promoting Muscle Regeneration by Modulating the Macrophage Phenotype

**Figure 2:**
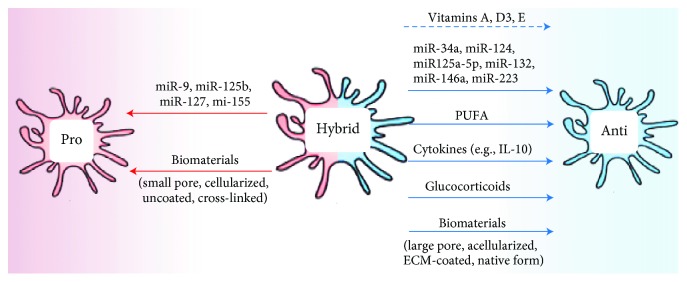
Macrophage-centered therapeutic approaches. Different strategies were developed to restore a balance in macrophage polarization in chronic degenerative muscle disorders. These strategies include cytokines (e.g., IL-10), nutritional compounds (e.g., PUFA and vitamins), RNA silencing (e.g., miRNA), pharmacological drugs (e.g., glucocorticoids), and biomaterials (synthetic, biological, or mixed). These strategies could be used to skew macrophage polarization toward their pro- or anti-inflammatory phenotype depending on the desired therapeutic effect.

Considering the detrimental effect of inflammation in dystrophic muscles, anti-inflammatory drugs are a standard therapeutic approach for many muscle diseases. Accordingly, glucocorticoids are the only drugs that consistently demonstrated efficacy on the preservation of muscle force and ambulatory function in DMD patients [106]. Glucocorticoid treatment reduces macrophage accumulation and promotes their switch toward the anti-inflammatory phenotype, which is correlated with reduction of muscle necrosis and preservation of muscle force and function in DMD [107, 108]. However, glucocorticoids are nonspecific and have many detrimental side effects. Particularly, they stimulate signalling pathways involved in muscle catabolism and indirectly contribute to muscle wasting [109]. Therefore, novel therapeutic approaches specifically targeting macrophages in order to restore their polarization are a promising avenue for the treatment of DMD (Figure 2) [110].

### 7.1. Anti-Inflammatory Cytokines and Growth Factors

#### 7.1.1. Interleukin-10

IL-10 has been used as an immune-based intervention because of its potential to deactivate proinflammatory macrophages and induce the anti-inflammatory phenotype *in vitro* [111–116]. To determine the role of IL-10 *in vivo*, the regenerative capacity of IL-10-null mice was investigated after hind limb muscle unloading and reloading [117]. The authors showed that IL-10 mutant mice exhibit high levels of proinflammatory markers (IL-6 and CCL2), persistent signs of muscle damage, and reduced accumulation of anti-inflammatory macrophages (expressing CD163 and arginase-1), leading to altered muscle regeneration [117]. Similarly, ablation of IL-10 expression in 12-week-old dystrophic mice reduces anti-inflammatory M2c macrophage polarization and muscle strength [118]. *In vitro* coculture assays revealed that IL-10 does not affect directly myoblast proliferation or differentiation, but rather affects myogenesis indirectly by promoting the transition of macrophages toward their anti-inflammatory M2c phenotype, which favours myoblast differentiation [117, 118]. Therefore, IL-10 has been considered as a therapeutic target to improve muscle regeneration; however, administration of IL-10 early in the regenerative process leads to the premature differentiation of myoblasts which reduces fiber size at 7 days postcardiotoxin injury [42] and may promote tissue fibrosis [118, 119].

#### 7.1.2. Insulin Growth Factor-1

IGF-1 is a key growth factor involved in numerous biological processes. During muscle regeneration, IGF-1 was shown to mediate myogenic cell proliferation, differentiation, and survival, and it also plays a crucial role in shaping the macrophage activation state [58, 120]. During muscle regeneration, IGF-1 is secreted by various cell types, including pro- and anti-inflammatory macrophages, which show a similar level of expression of this growth factor [58]. Conditional deletion of the IGF-1 gene in myeloid cells promotes the accumulation of the Ly6C^hi^ proinflammatory monocyte/macrophage phenotype and reduces CD206^+^ anti-inflammatory macrophages during muscle regeneration, leading to increased fat deposition and reduced fiber size at 10 days after cardiotoxin injury [58]. Analysis of the transcriptional profile showed that IGF-1 deletion skewed macrophages toward their proinflammatory profile, which indicates that IGF-1 is an autocrine factor regulating macrophage polarization [58]. Moreover, IGF-1 is necessary for IL-4-induced transition of macrophages toward their anti-inflammatory phenotype [121]. The therapeutic efficacy of IGF-1 injection has been observed by increased fiber size after sterile muscle injury in transgenic mice [45, 122] and improved muscle strength in old adult mice [123]. However, because myeloid cell-derived IGF-1 peaks by 3 days postinjury and then decline to baseline [45], the long-term beneficial effect of IGF-1 remains uncertain. Particularly, since IGF-1 is involved in a variety of cellular processes, its exogenous administration could have detrimental side effects. For instance, IGF-1 suppresses circulating insulin and growth hormone levels, causing hypoglycemia in humans [124] and stimulates human osteogenic sarcomas [125].

Overall, anti-inflammatory cytokines and growth factors have a great potential to skew macrophages toward their anti-inflammatory profile, which would be beneficial in chronic degenerative muscle diseases; however, the mitigation of their potential side effects is technically challenging and remains a concern for the development of successful therapeutic approaches (Table 1).

### 7.2. RNA Silencing

#### 7.2.1. Small Interfering RNA

The potential of small interfering RNA (siRNA) has been investigated in many studies to silence proinflammatory markers and adhesion molecules such as TNF-*α*, VCAM-1, and P-selectins during inflammatory diseases [126, 127]. The ability of siRNA to promote macrophage skewing toward their anti-inflammatory phenotype has been evaluated in different conditions. For instance, the delivery of siRNA targeting collapsin response mediator protein-2 (CRMP2) through lipidoid nanoparticles resulted in a drastic switch toward the anti-inflammatory macrophage phenotype which decreased inflammation, fibrosis, and heart failure postmyocardial infarction [128]. Similarly, silencing of TIMP-1 in proinflammatory macrophages was shown to promote their proangiogenesis capacity at a similar level than the anti-inflammatory phenotype [77]. Receptors such as toll-like receptor-4 (TLR4) and CCR2 are also potential targets in inflammatory diseases and healthy muscle regeneration. The ablation of these receptors blunts the total macrophage accumulation, which results in reduced inflammation in acute injury and dystrophic mdx mice [45, 129, 130]. Genetic or pharmacologic blockage of TLR4, TLR2, or CCR2 in chronic degenerative muscles of mdx mice reduced total macrophage numbers and skewed them toward an anti-inflammatory profile (iNOS^−^CD206^+^), leading to enhanced histopathology and muscle force generation [94, 129, 131]. On the other hand, loss-of-function of TLR2 or CCR2 decreases total monocytes/macrophages during acute muscle injury but fails to polarize macrophages toward an anti-inflammatory phenotype and causes abnormal persistence of necrotic fibers and impaired regeneration [44, 45, 131]. Therefore, the therapeutic strategies aiming at reprograming the macrophage phenotype must be carefully selected for the treatment of chronic degenerative conditions, since the controlled and coordinated accumulation of both pro- and anti-inflammatory phenotypes is essential for optimal muscle healing after an acute injury.

#### 7.2.2. Small Noncoding RNA Molecules

MicroRNA (miRNAs) are small noncoding RNA molecules containing about 22 nucleotides, which function as posttranscriptional regulators of many genes and cellular processes in an autocrine or paracrine manner. Multiple miRNAs were shown to be involved in macrophage polarization. For instance, miR-9, miR-127, miR-155, and miR-125b were classified as proinflammatory inducers, while miR-124, miR-223, miR-34a, let-7c, miR-132, miR-146a, and miR-125a-5p skewed macrophages toward an anti-inflammatory phenotype [132]. The way that miRNAs regulate proinflammatory macrophage phenotypes includes silencing of specific targets such PPAR-*δ*, B-cell lymphoma 6 (Bcl6), dual-specificity protein phosphatase 1 (Dusp1), signal transducer and activator of transcription-6 (STAT6), C/EBP, suppressors of cytokine signalling-1 (SOCS1), and interferon regulatory factor 4 (IRF4) and stimulating the c-Jun N-terminal kinase (JNK) pathway [133–135]. miRNAs that promote anti-inflammatory polarization mechanistically inhibit Notch1, signal-regulatory protein beta-1 (SIRPb1), STAT3, C/EBP-*δ*, and interleukin 1 receptor-associated kinase-1/tumour-necrosis-factor-receptor-associated factor-6 (IRAK1-TRAF6) [136–138]. Transfection of either miR-34a, miR-146a, or miR-132 reduces the levels of proinflammatory-associated markers (iNOS, IL-12) upon LPS challenge [136, 137] and enhances anti-inflammatory markers [137]. Knockout gene strategies of either let-7c or miR-124 also showed an increase in proinflammatory markers (CD86, iNOS, TNF-*α*, and IL-12), in parallel with a decrease in anti-inflammatory-associated markers (FR-b, CD206, and Ym1) [136, 139]. Delivering a mixture of miR-1, -133, and -206 after laceration of rat tibialis anterior muscle enhances muscle regeneration and prevents fibrosis formation [140]. Although the effect of these myomiRNAs is likely to be mediated through a direct muscle-specific effect, rather than by acting on inflammation, it illustrates the potential of this therapeutic strategy for muscle disorders. However, the use of miRNA as a therapeutic approach is challenging because of inappropriate biodistribution, poor *in vivo* stability, and untoward side effects (Table 1) [141].

### 7.3. NF-*κ*B Inhibitors

NF-*κ*B is a key transcription factor in macrophages that is required for the expression of numerous proinflammatory genes [142]. In DMD, the NF-*κ*B pathway is persistently overexpressed in immune cells and skeletal muscle cells [143]. Inhibition of this pathway specifically in myeloid cells of dystrophic mdx mice reduced inflammation and muscle necrosis, while its specific deletion in muscle progenitor cells increased myogenesis [143]. Pharmacological inhibition of this pathway mitigated the disease and improved muscle function in dystrophic mdx mice and golden retriever muscular dystrophy dog model [143, 144]. Therefore, it is a promising therapeutic target for chronic muscle diseases.

However, while the NF-*κ*B pathway has been initially described as an inflammatory pathway, accumulating evidence indicate that its effect on inflammation is more complex than anticipated [145]. A pioneer study showed that the inhibition of NF-*κ*B during the onset of inflammation reduces the inflammatory response, while its inhibition during the resolution of inflammation results in the prolongation of the inflammatory response [146]. *In vitro* models have also shown that inhibition of NF-*κ*B impairs the maturation of human monocytes into both pro- and anti-inflammatory macrophages [147]. Other models have shown that the effect of the NF-*κ*B pathway varies depending on different factors such as the type of cell and insult. For instance, in a model of bacterial infection, the specific knockout of IKK*β* (factor involved in the NF-*κ*B pathway) in airway epithelial cells inhibited inflammation; however, its inhibition in myeloid cells promoted the inflammation response [148]. IKK*β*-deficient macrophages showed increased markers of inflammation and an impaired ability to skew to their anti-inflammatory phenotype, which suggests an important role of NF-*κ*B in the macrophage phenotype switch [148]. Overall, while NF-*κ*B inhibitors are attractive compounds for the treatment of chronic muscle disorders, the broad and complex roles of this pathway make it a difficult target for the development of a macrophage-centered therapeutic approach (Table 1) [145].

### 7.4. Nutritional Compounds

#### 7.4.1. Proteins and Amino Acids

Many nutritional compounds were shown to regulate the inflammatory process, which represent a simple therapeutic approach for the treatment of chronic muscle disorders. Cod and shrimp proteins were shown to decrease the density of neutrophils and proinflammatory macrophages, while increasing the anti-inflammatory subset in rat muscles following acute sterile injury [149–151]. The beneficial effects of cod protein on the resolution of inflammation and muscle regeneration after injury were attributable to its high content of arginine, glycine, taurine, and lysine [150]. These amino acids have been shown to decrease muscle cell damage in various rodent models of inflammation including endotoxin- and exercise-induced muscle damage by inhibiting the secretion of inflammatory markers, such as TNF-*α*, IL-1*β*, IL-6, and PGE_2_, and by reducing COX-2 expression and ROS generation [152–158]. The protective effects of L-arginine on muscle cell membrane integrity in mdx mice was reported to be mediated through a decrease in TNF-*α*, IL-1*β*, and IL-6 expression levels [153]. Therefore, dietary fish protein rich in arginine, glycine, and taurine represents a safe, inexpensive, and efficient approach for the treatment of inflammatory musculoskeletal diseases (Table 1).

#### 7.4.2. Long-Chain Polyunsaturated Fatty Acids

Omega-3 polyunsaturated fatty acids (PUFA) were shown to have a variety of anti-inflammatory effects such as decreasing adhesion molecules and leukocyte chemotaxis in a variety of inflammatory conditions [159–161]. This effect is partly mediated by their ability to inhibit NF-*κ*B-dependent inflammatory genes and blunt the production of eicosanoids, such as prostaglandins and leukotrienes [162]. In addition to reducing leukocyte accumulation, PUFA directly target macrophages to inhibit their activation and promote their switch toward their anti-inflammatory phenotype [163]. In skeletal muscle, the long-term therapy of omega-3 supplementation to mdx mice reduced proinflammatory markers (TNF-*α* and NF-*κ*B levels) and improved muscle regeneration [164]. Similarly, a diet supplemented with fish oil diminished the signs of inflammation and reduced fibrosis in the diaphragm muscle of old mdx mice [165]. Therefore, a diet rich in PUFA represents a simple strategy for the treatment of chronic muscle disorders.

#### 7.4.3. Vitamins and Antioxidants

Different studies looked at the role of vitamins to regulate inflammation and macrophage phenotype. So far, retinoic acid (active form of vitamin A), vitamin D3, and vitamin E have been shown to play a role in the functional polarization of macrophages. Using a microarray to scan over 40,000 genes in peritoneal macrophages, it was shown that retinoic acid acts through GATA-6 signalling to change the profile of macrophages, which acquire some markers of the anti-inflammatory profile (e.g., Arg1) but not others (e.g., CD206) [166]. These findings show that retinoic acid promotes an anti-inflammatory-oriented profile that is located in a broad spectrum of macrophage polarization states. Retinoic acid was also shown to potentiate the ability of IL-4 to skew macrophages toward their anti-inflammatory phenotype, indicating that macrophage polarization is a result of the complex interaction of various molecular components [167]. Skeletal muscle regeneration was delayed in mice deficient in retinoic acid receptor-*γ*, while the treatment of injured wild-type mice with a retinoic acid receptor-*γ* agonist reduced fibrotic/adipose tissue and improved muscle repair [168]. However, the exact contribution of macrophages in the positive effect of retinoic acid on skeletal muscle repair remains to be determined.

Vitamin D3 has an inhibitory role in a plethora of cellular immune processes, including in T cells, by reducing the inclination of Th0 toward Th1 cells, along with a selective reduction of Th1-related cytokines [169, 170]. Moreover, vitamin D3 was shown to promote Treg development, which plays an important role in driving the M2 macrophage phenotype [171]. Vitamin D3 deficiency was shown to impair the maturation of monocytes to macrophages, while vitamin D3 addition increases the expression of macrophage-specific surface antigens. Macrophages treated with vitamin D3 adopt an intermediary phenotype located on the broad spectrum of macrophage polarization, which is characterized by a controlled increase in oxidative burst, chemotaxis, and phagocytosis, together with a decrease in the expression of TLR2/4 and a reduced level of the proinflammatory cytokines TNF-*α*, IL-1, and IL-6 [172].

As the most abundant lipid soluble chain-breaking antioxidant in cell membranes, vitamin E has been shown to prevent mitochondrial oxidative damages and entrap peroxyl radicals and oxygen species, all of which are putative factors in several human diseases [173]. Besides its well-known antioxidant properties, accumulating evidences support the immunostimulating effects of vitamin E in pathogen-infected subjects through different mechanisms that enhance the Th1-like pattern immune response [174]. In conditions with a low-grade inflammation (e.g., obesity and aortic lesions), vitamin E appears to suppress infiltrating macrophage accumulation and related cytokines [175, 176]. Indeed, *γ*-tocopherol, one of the active forms of vitamin E, substantially reduced the recruitment of adipose tissue macrophages in high-fat-fed mice. Moreover, LPS-mediated proinflammatory macrophage polarization was reduced in *γ*-tocopherol-treated human adipose tissue with minimal influence on alternative polarization into anti-inflammatory macrophages [175].

Altogether, these findings indicate that vitamins are not classical inducers of the anti-inflammatory phenotype, but they rather promote an intermediary phenotype located in the continuum of macrophage polarization. Thus, the contribution of vitamins to the promotion of the pro- or anti-inflammatory phenotype of macrophages is dependent on their combinatory effect with other molecular and cellular components.

### 7.5. Biomaterials

The advancement in bioengineering led to the development of new implantable medical devices that can be used in regenerative medicine to modulate macrophage response in different tissues, such as skeletal muscles [177]. The interface between the biomaterial surface and the tissue initiates cellular events that activate a subsequent signalling cascade of paracrine and autocrine factors in the host tissue. These biomaterials can be either synthetic (biodegradable or nonbiodegradable) or biologic [178, 179].

#### 7.5.1. Biologic Materials

These biomaterials include human and porcine skin substitutes, porcine small intestine submucosa, dermal, and other natural substitutes (e.g., collagen, chitosan, silk, and keratin) [178, 180]. The nature and the age of the source animal have a significant impact on the effect of the transplanted biomaterial. For instance, porcine small intestine submucosa harvested from pigs at different ages revealed that a scaffold isolated from younger animals promote a dominant anti-inflammatory macrophage response and better muscle regeneration than a scaffold derived from older animals [181]. The macrophage response is also affected depending on whether the scaffold is implanted in its native form or its cross-linked form (which increases the protein cross-links to improve stability and durability), the former enhancing the anti-inflammatory phenotype, while the latter promoting the proinflammatory phenotype of macrophages [182, 183].

#### 7.5.2. Synthetic Biomaterials

Synthetic biomaterials such as polyethylene, polyethylene terephthalate, polyacrylamide, perfluoropolyether, and polydioxanone elicit an anti-inflammatory response in macrophages *in vitro* [178]. Macrophage response to biomaterials is dependent on many factors including their composition, characteristics (dimension, pore size, and topography), and the quality of the sterilisation [178]. The pore size is a critical regulator of macrophage polarization, with a smaller pore size inducing the proinflammatory phenotype of macrophages cultured on perfluoropolyether [184], while larger pores induce an anti-inflammatory response [185]. In addition to pore size, other factors such as the nature of the material play a significant role in polarizing macrophages, since macrophages cultured on expanded polytetrafluoroethylene and chitosan with large pores show a proinflammatory cytokine profile [186, 187].

#### 7.5.3. Hybrid Biomaterials

These biomaterials are derived from both synthetic and biologic materials. For instance, the coating of polypropylene mesh with ECM components (isolated from decellularized porcine skin) was shown to increase the ratio of anti-inflammatory macrophages compared to uncoated polypropylene mesh [188]. Moreover, these biomaterials could be used as a carrier for biochemical cues (e.g., cytokines and growth factors) or pharmacological compounds. Therefore, biomaterials could be used as a mixed therapy with other anti-inflammatory-stimulating factors described previously. Moreover, biomaterials can also be used as a carrier in cellular transplantation experiments (e.g., for macrophages, satellite cells, or other stem cells). For instance, a tissue engineering strategy showed that a compound containing mesenchymal stem cells and a decellularized ECM scaffold synergistically promoted macrophage polarization toward the M2 phenotype and improved skeletal muscle regeneration in rats [189]. Biomaterials were also shown to improve the success of myoblast transplantation; however, the contribution of macrophage polarization in the beneficial impact of these biomaterials is still elusive [190].

Biomaterials were also used as a scaffold to increase muscle regeneration. Acellular biological scaffolds were shown to elicit an anti-inflammatory macrophage response resulting in constructive remodelling, while scaffolds containing cellular components were associated with a proinflammatory macrophage response resulting in fibrosis and failed regeneration [191]. Biomaterials were also tested as a strategy to improve innervation, vascularization, and myofiber contractility in skeletal muscles [192]; however, the potential of biomaterials as a macrophage-centered approach for the treatment of DMD remains to be investigated. Nonetheless, the recent advances in bioengineering open an exciting new therapeutic avenue that could be used in combination with other factors regulating macrophage polarization for the treatment of chronic degenerative muscle disorders (Table 1).

### 7.6. Macrophage Transplantation

Transplantation of M2 macrophages is considered as a new cell-based therapy for many diseases including Alzheimer, diabetes, and peripheral arterial disease [193–195]. In a rat model of Alzheimer, M2 macrophage transplantation greatly attenuated inflammation and cognitive impairment by skewing endogenous microglial cells toward the M2 phenotype [193]. Moreover, systemic administration of peritoneal M2 macrophages enhanced glucose tolerance, prevented rejection, and prolonged the survival time of islet allografts in diabetic mice [194]. With regard to skeletal muscle regeneration, it was shown that transplantation of M1-polarized macrophages (LPS/IFN-*γ*) following ischemia-induced muscle injury enhanced the recovery of muscle function, while the administration of nonpolarized macrophages did not [196]. Similar results were observed in another model of muscle injury (laceration) [96]. Another study demonstrated that early administration of M1-polarized macrophages (IFN-*γ*) reduced fibrosis and improved myofiber size and muscle function, while early administration of M2-polarized macrophages (IL-4/IL-13) improved myofiber size but not muscle force and fibrosis [195]. These results indicate that the transplantation of macrophages needs to be timely coordinated to improve skeletal muscle regeneration. Notably, the safety and efficacy of macrophage transplantation have already been tested in two clinical studies, showing a significant improvement of motor and cognitive activities in patients with stroke and neurological affectations [197, 198].

Satellite cell transplantation is also a promising therapeutic avenue to treat different muscle diseases; however, it faces many technical challenges such as poor cell survival, lack of self-renewal, and long-term engraftment. Macrophages represent an attractive approach to improve the success rate of satellite cell transplantation. For instance, coinjection of myoblasts with proinflammatory macrophages supported myoblast engraftment by extending their proliferative phase and delaying their differentiation, while coinjection with anti-inflammatory macrophages did not improve myoblast engraftment [199]. Altogether, these findings suggest that macrophages are an interesting therapeutic approach, either as a direct therapy or as a cofactor for the transplantation of other cell types. However, macrophage polarization needs to be tightly regulated to optimize muscle regeneration.

## 8. Conclusion

Muscle regeneration relies on different stem cell types, especially satellite cells and FAPs. While these cells are the ultimate executors of muscle repair, their activity is regulated and coordinated by neighbouring cells. Particularly, macrophage polarization toward their proinflammatory or anti-inflammatory phenotype has been shown to play key roles in myogenesis and skeletal muscle healing. The novel insights into the field of inflammation have revealed that macrophages span a continuum of polarization states, which evolves depending on intrinsic and extrinsic factors. In chronic degenerative muscle disorders, the abnormal phenotype adopted by macrophages was shown to contribute to this detrimental process. Therefore, new therapeutics targeting macrophage polarization such as cytokines and growth factors, nutritional compounds, RNA silencing, pharmacological drugs, and biomaterials are tested to improve skeletal muscle regeneration. Depending on the type of muscle injury and on the desired therapeutic effect, these strategies could be used to skew macrophage polarization toward the proinflammatory phenotype (e.g., to decrease excessive fibrosis) or toward the anti-inflammatory phenotype (e.g., to dampen inflammation and promote myogenesis). Despite some technical challenges, these new strategies have a strong therapeutic potential to mitigate different muscle disorders such as DMD. The recent technological advances combined with our improved comprehension of the role of macrophages in skeletal muscle regeneration and diseases will synergize to develop this promising field of research.

## Figures and Tables

**Table 1:** Table showing the pros and cons of the different therapeutic approaches targeting macrophages to improve muscle regeneration and/or mitigate muscle diseases.

Therapeutic approaches	Advantages	Challenges	References
Anti-inflammatory cytokines (e.g., IL-10)	Endogenous molecules; deactivate proinflammatory macrophages and induce the anti-inflammatory phenotype	Short-term effect; nonspecific (could directly impair other cellular processes in skeletal muscle regeneration)	[115, 117]
Growth factors (e.g., IGF-1)	Endogenous molecules; promote macrophage transition to their anti-inflammatory phenotype; promote muscle growth	Short-term effect; systemic side effects	[121, 124, 125]
RNA silencing (e.g., miRNA, siRNA)	Specifically target genes implicated in chronic inflammation; skewed macrophages toward pro- or anti-inflammatory phenotype	Poor stability; inappropriate distribution; off-target side effects; delivery	[126, 127, 136, 138, 200]
NF-*κ*B inhibitors	Dampen inflammation; easy to deliver; good stability	Nonspecific (could directly impair other cellular processes in skeletal muscle regeneration)	[143]
Nutritional compounds (proteins, amino acids, PUFA, vitamins, and antioxidants)	Promote macrophage transition; potentiate the effect of other therapies; inexpensive; easy to administer	Mild therapeutic effect	[166, 171, 172]
Biomaterials	Skewed macrophages toward pro- or anti-inflammatory phenotype; local effects; long-term effects; combination with other therapies	Invasive; biocompatibility; risk of contamination; degradation of the biomaterial	[201]
Macrophage transplantation	Specifically deliver the desired macrophage subset; increase the success rate of satellite cell transplantation	Invasive; systemic side effects; expensive; time consuming	[193–195]
